# The Development of Novel Cu/GO Nano-Composite Coatings by Brush Plating with High Wear Resistance for Potential Brass Sliding Bearing Application

**DOI:** 10.3390/ma17112623

**Published:** 2024-05-29

**Authors:** Yingdi Feng, Xiaoying Li, Hanshan Dong

**Affiliations:** School of Metallurgy and Materials, University of Birmingham, Birmingham B15 2TT, UK; h.dong.20@bham.ac.uk

**Keywords:** electron brush plating, Cu/GO composite coatings, tribological properties, brass sliding bearing

## Abstract

Low friction and high wear resistance are critical properties for sliding bearings. In this research, advanced Cu/GO nanocomposite coatings have been developed by a brush plating method to improve the tribological performance of brass-based sliding bearings. A series of brush plating studies under voltages from 2 to 6 V with different GO concentrations (0.2–0.8 g/L) was conducted, and the coating microstructures were characterised by SEM, EDX, GDOES and XRD and the tribological behaviour of the Cu/GO composite coatings were evaluated using dry ball-on-plane tribological tests The experimental results have demonstrated that GO can be successfully introduced into the whole composite coating layer; the Cu/GO composite coatings can reduce the friction of brass and increase its wear resistance by two orders of magnitude, mainly due to the self-lubricating GO added into the coatings.

## 1. Introduction

Brass is the most common lead-free alloy used to manufacture sliding bearings [1,2]; however, it possesses moderate tribological properties. Hence, conventional lubricants are required, such as oil, but this presents environmental concerns. Therefore, research into self-lubricating parts with high wear resistance is attracting more and more attention.

Graphene has become a hot research area in recent years, mainly for electronic and optoelectronic applications. However, limited research highlights the tribological property improvement of a few layers of graphene (Gr) in terms of reducing friction and wear of tribological pairs (such as bearings). It has been found that graphene could present as good lubricity as bulk graphite on a micro-scale [3]. Therefore, a few layers of graphene were researched as self-lubricating coatings for engineering materials. However, the very low interface adhesive strength and thinness of the coating have limited the stability and durability of graphene coatings for industrial applications.

There is research focused on the solid lubricant properties and wear resistance enhancement of copper (Cu) nanoparticles [4,5,6] by adding Cu particles into oil-based lubricants or adding graphene oxide (GO) particles, with mechanical bonding to each other. Only limited layer thickness and relatively weak bonding to the substrate hindered the application, especially in the service life [7,8]. How to produce a self-lubricant coating on brass with sufficient layer thickness and strong bonding to the substrate presents both a technological and scientific challenge.

As graphene oxide (GO) possesses great hydrophilicity due to the distribution of a large number of polar oxygen-containing functional groups on the surface of the graphene oxide, it can be dissolved into aqueous-based electrolyte solutions. Moreover, the electronegative property presented in aqueous solutions during brush plating enables GO movement within an electric field. Hence, applying GO-containing coatings by electrochemical deposition techniques is possible, in principle [9]. There are mainly two electro-deposition techniques to produce GO coatings, which are electro-plating deposition (EPD) [10,11,12] and electro-brush plating (EBP) [13,14].

The conventional electro-plating deposition (EPD) technique produces a GO/metal composite coating by deposition of positively charged metal ions (such as Ni ions in Ni plating) together with GO to the substrate (cathode) [11,12]. The bonding introduced by the electric field is stronger than the graphene coatings produced by solution-flow or CVD transferring methods. However, the agglomeration of the GO particles in aqueous solutions, such as CuSO4 solution, will significantly reduce the uniformity of GO in the GO/metal composite coating with aggregated GO particles mainly enriched on the surface [15,16]. This problem could be addressed by EBP mainly because the continuous brush movement can improve the uniformity of the GO suspension in Ni plating solution, as reported in [17].

However, no work has been reported on the development of graphene/copper nanocomposite coatings using electro-brush plating (EBP). Therefore, the current research was directed at developing novel Cu/GO composite coatings by the electro-brush plating technique with low friction and high wear resistance for potential sliding-bearing applications. To this end, a series of EBP processes were conducted to study the effect of the processing parameters and GO concentration in plating solution; the coating microstructures were characterised and their tribological properties were evaluated to advance scientific understanding and for the optimisation of the EBP processing conditions for optimal Cu/GO coatings for potential brass sliding bearing applications.

## 2. Materials and Methods

### 2.1. Substrate Materials and Coating Solutions

Grade CZ121M brass rod, purchased from RS Components Ltd. (Fort Worth, TX, USA), was used as substrate material and the composition was Cu 57–59%, Pb less than 0.1% and Zn as the balance. Brass rods of 21 mm in diameter were cut to 5 mm in thickness. Those discs were ground to #1200 with SiC paper before brush plating. Soluble graphene oxide powder was supplied by SIGMA-ALDICH, Co., Ltd. (Dorset, UK). A total of 0.020 g of GO powder was dispersed in 100 mL Cu acid brush plating solution and then the mixture was suspended for 1 h in an ultrasonic bath (200 W, Ultrawave U2500H) before plating. Active, clean liquid and Cu plating solutions were obtained from SPA Plating Co., Ltd. (Bath, UK). The nickel strike plating solution was supplied by SIFCO Applied Surface Concepts (ASC) Ltd. (Birmingham, UK).

### 2.2. Cu and Cu/GO Coating Processes

Cu and Cu/GO coatings on brass were produced by brush plating using MF Rectifier MK II (SPA Plating Co., Ltd.). The brush plating process consists of three main steps: active cleaning, nickel strike and Cu or Cu/GO coating. The active cleaning process was used to remove oxides on the brass surface, while the nickel strike process aimed to improve the adhesive strength between the brass substrate and the coating layer. After every step, the sample surface was put under cold running water to remove the remained plating solution, rinsed with deionised water for 1 min and finally dried with hot wind. The detailed process parameters are depicted in Table 1.

The plating processes were designed into two groups to identify the optimal brush plating parameters. Firstly, brush plating was carried out with Cu or Cu + GO (0.2 g/L) solutions under the plating voltage of 2 to 6 V, as listed in Table 2 for Group 1 samples, to study the effect of the plating voltage. Based on the microstructure characterisation and property evaluation, the optimal plating voltage (5 V), which produced the coating samples with the best tribological performance of this group, was selected for the second set of brush plating investigations. The solution used for the second group of brush plating contained GO concentrations ranging from 0.2 to 0.6 g/L, as detailed in Table 2. For Group 1 samples, there are two subgroups: one is coated using Cu plating solution only and the other is Cu with GO (0.2 g/L) solution. The sample code, the corresponding brush plating conditions, and the solution composition are summarised in Table 2.

### 2.3. Characterisations of Coating Structures

The surface morphology and worn tracks of as-plated specimens were observed by Joel 7000 Field Emission Scanning Emission Microscope (SEM). To obtain the cross-sectional view of coating layers, specimens were cut and mounted for metallurgical sample preparation. Then, the samples were ground on SiC abrasive paper from grit 120 to 1200, polished with a 1 µm diamond suspension and etched with acidic ferric chloride for 20–30 s. Apreo 2S HiVac SEM with colour-chemi EDX [18] was used to observe the surface morphology, cross-sectional layer structure, worn counterpart and chemical compositions. The surface layer thickness was measured during SEM observation on the cross-section layer structures and the average of three measurements was given. The depth distribution of carbon content along the coating layer was measured by GDOES (SPECTRUMA GDA-650HR).

XRD was performed by Proto AXRD to scan the surface region of the specimens with a scanning step of 0.015°/s from two theta, 20° to 100° using Cu-Kα radiation source (λ = 0.15419 nm). PANalytical X’Pert Highscore Plus software V3.0 was used to identify the phases.

To identify the graphene oxide after the tribological test, Raman spectroscopy Renishaw inVia Reflex was used to identify the oxides after wearing tests. The worn surface of GO/Cu coated samples was placed on a flat microslide which was focused with a “20×” objective microscope. Under a 488 nm wavelength laser source, Raman spectra were obtained from 100 to 900 cm^−1^ and 1000 to 3500 cm^−1^. It used 10 s of exposure time from a 35 W laser source and three times the accumulation for each scan. WIRE software (version 4.2) produced by Renishaw was used to process the data and the Raman peaks were identified by referring to the data in the literature.

### 2.4. Tribological Test

The tribological behaviours of the substrate brass, Cu and CuGO coatings against ϕ8 mm 316 stainless steel balls (purchased from TN United Kingdom Ltd., West Sussex, UK) were performed under room temperature (approximately 18 °C) and approximately 47% humidity (MT-903, ATP Instrumentation Ltd., Leicester, UK) with a ball-on-plane tribometer (TE79, Phoenix Tribology Ltd., Oxford, UK). The tests were carried out under a 5 N load at a speed of 5 mm/s at 0.5 Hz frequency. Each test lasted for 30 min. Before the tribological test, the steel balls were cleaned with an ultrasonic bath in soapy water and then with acetone to remove organic pollutants. The counterpart was replaced after one complete test, and a new surface on the counterpart was used in repeated tests (at least 3 times) to obtain a relatively reliable tribological data.

Surface hardness was measured using an MKV-H1 hardness tester (Mitutoyo, Kawasaki, Japan) with a Vickers indenter under the load of 10 g and the hardness value was an average of five measurements. Surfcorder SE1700 profilometer was used to evaluate the wear performance of the coating samples and the uncoated brass. The 2D worn area along the wear track was scanned and calculated in three positions. Then, the worn area multiplied by the wear track length 5 mm, determines the wear volume loss.

## 3. Results

### 3.1. Surface Morphology and Roughness

**Figure 1:**
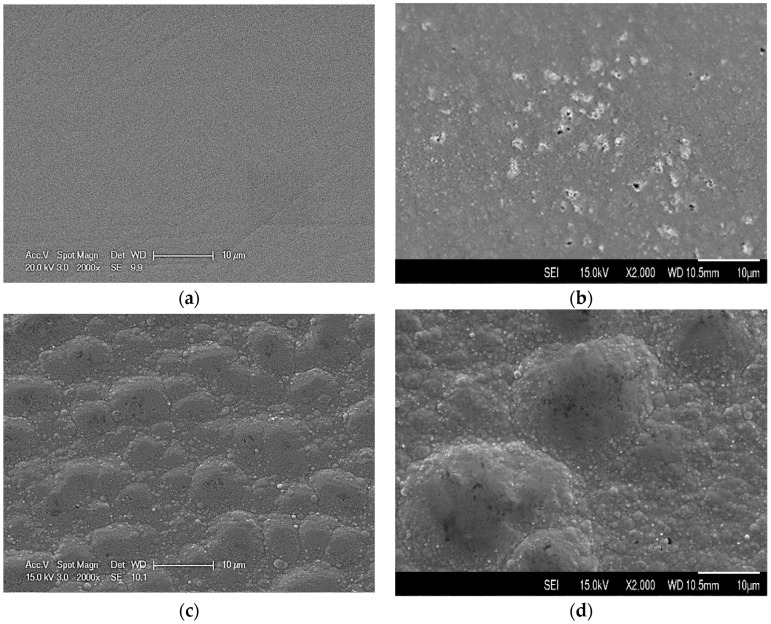
Surface morphology of coating samples: (**a**) Cu2; (**b**) CuGO2; (**c**) Cu5; (**d**) CuGO5.

Visual observations revealed that the surface colour of the brush-plated graphene oxide (GO) reinforced copper (Cu) composite coatings and copper coatings changed from yellow to red when increasing the plating voltage. When plated under 3 V, very smooth surfaces with fine granular features were observed for both Cu and CuGO samples, but some black spots were evidently seen on the CuGO surface, as shown in Figure 1a,b. Cauliflower-like structures were observed when increasing the plating voltage to over 4 V as exemplified in Figure 1c,d.

**Figure 2:**
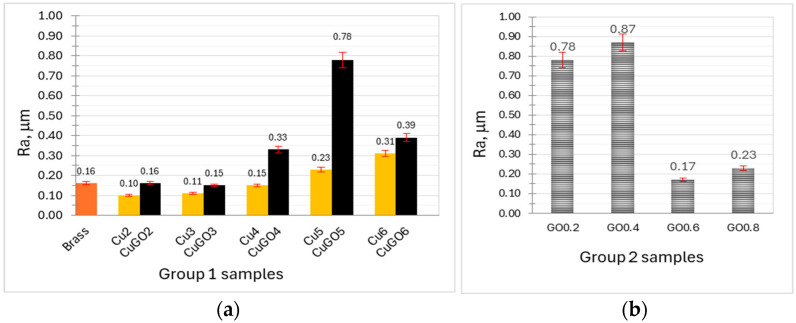
Surface roughness of (**a**) Group 1 and (**b**) Group 2 samples (see Table 1 for sample details), Ra of brass for comparison.

The surface roughness of all coated samples is charted in Figure 2, with the uncoated brass sample for comparison. It can be seen that, for Group 1 samples, the surface roughness gradually increased with the increase in the plating voltages, but one exception is for the CuGO5 sample, which showed the highest roughness of the group. The Cu coating samples are smoother than the CuGO coating samples when coated under the same voltage, and the Cu2 and Cu3 coatings are even smoother than the uncoated brass surface. Figure 2b depicts the Ra values for Group 2 samples, revealing the GO concentration effect on the surface roughness change in the samples coated under 5 V. It can be seen that the samples with GO concentrations of 0.2 and 0.4 g/L possess high Ra values of 0.78 and 0.87 mm, respectively, while further increase in the GO concentration of the Cu solution to 0.6 and 0.8 g/L led the decrease in Ra values to 0.17 and 0.23 mm, respectively.

### 3.2. Layer Structure of the Coatings

**Figure 3:**
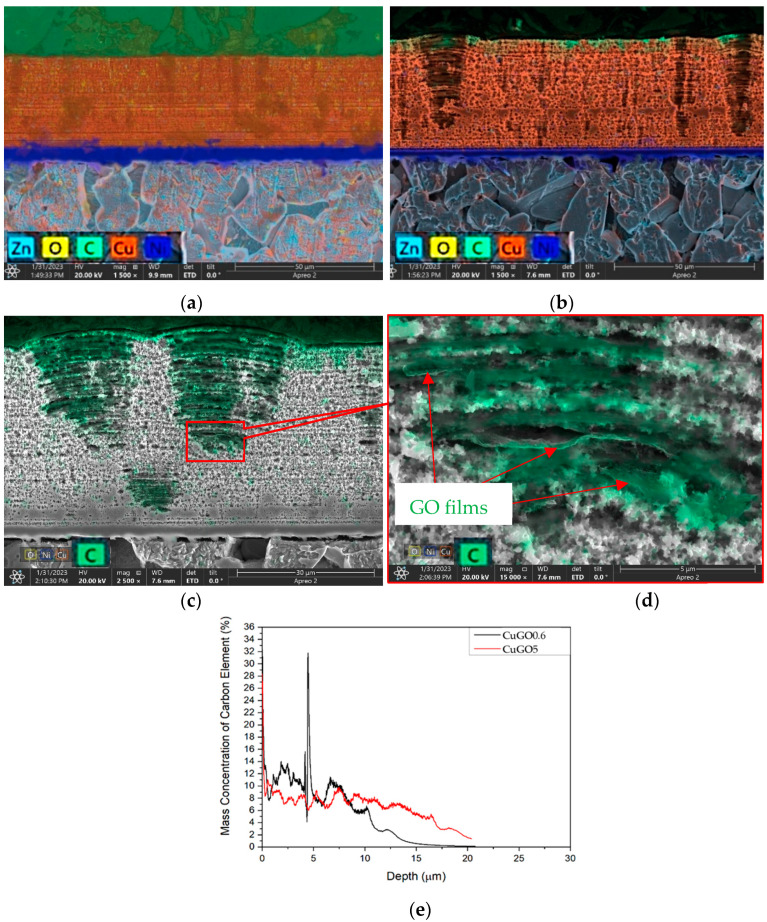
Typical SEM images of coating layer structures (**a**) Cu4 and (**b**) CuGO5 samples. (**c**,**d**) typical SEM images of well-developed columnar structures of CuGO samples showing GO within the lamellar layers. (**e**) GDOES depth profiles of carbon concentration, showing introduced GO content.

Typical cross-sectional layer structures of the Cu and CuGO coatings are shown in Figure 3a,b. It can be seen under the colour-chemi EDX combined with the SEM image with EDX mapping [18] that the coatings consist of two sublayers on the substrate: a Ni interface layer (in blue) and a lamellar Cu or CuGO top layer (in red-brown). The lamellar structure is the typical feature of the coatings plated by brush plating due to the repeated movement of the plating pen wrapped with an absorbent cloth with the electroplating solution on the surfaces. In addition to the horizontal lamellar structure, a vertical column structure could be observed when coatings were processed under voltages above 4 V, especially for CuGO samples. It can be also seen from Figure 3b that some of the columns are protruded where the cauliflower-like structures are observed on the surface (Figure 1b). The columnar width is about 5–20 mm, which corresponds to the diameter of the cauliflower head, as shown in Figure 1c,d. SEM observation and EDX composition analysis of the CuGO samples confirmed that GO was successfully embedded in the lamellar layers, and the GO films were concentrated in the well-developed columnar structures, as evidenced in Figure 3c,d. The GDOES depth profiles of two CuGO samples (Figure 3e) showing carbon contents of 6–14 wt% within the coating layers further confirmed the successful introduction of the GO to the plated CuGO coatings. The fluctuation of the carbon contents demonstrated the distribution of GO films within the lamellae layers. A sharp peak of high carbon content presented for the high GO content sample implied agglomerated GO films within the layer, which will reduce the coating density and wear resistance (see details in Section 4.2).

**Figure 4:**
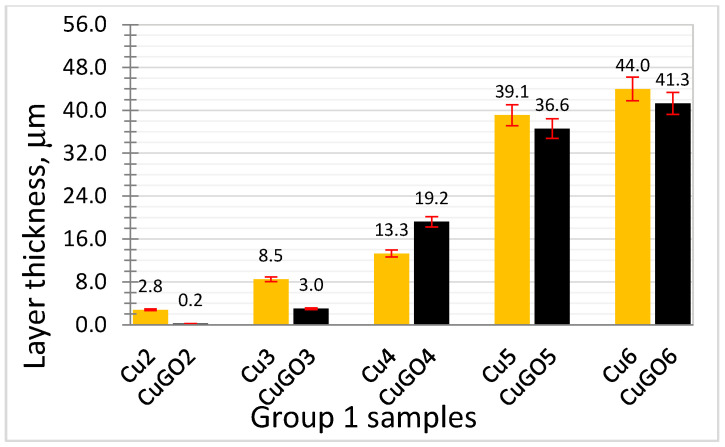
Cu and CuGO coating layer thickness under processing voltages of 2–6 V.

Figure 4 depicts the layer thickness of Group 1 coating samples. It can be seen that the layer thickness increases with increasing plating voltage, with a fast increase for 5 V and 6 V plated coatings. When coated at voltages of 2 and 3 V, very thin CuGO layers were formed. The coating layer thickness of the Group 2 samples was similar to that of the CuGO5 sample, indicating that under the same processing voltage (and time), the GO concentrations have limited, if any, effect on the layer thickness.

**Figure 5:**
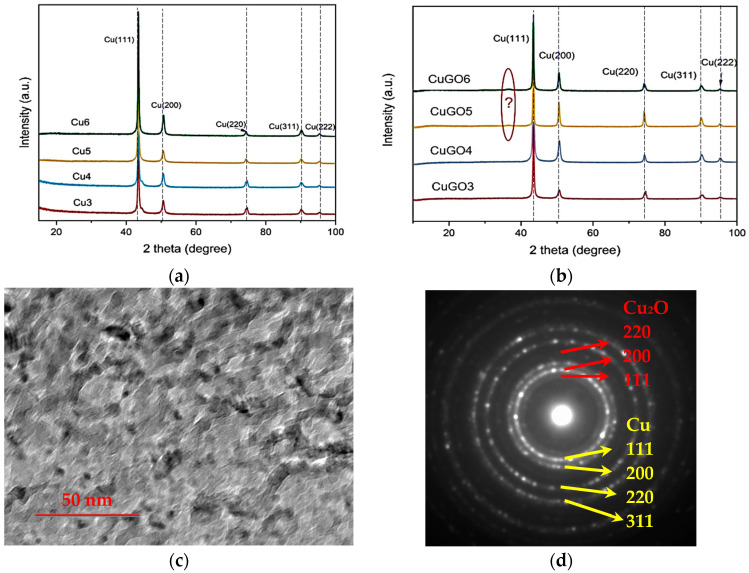
XRD patterns of (**a**) Cu and (**b**) CuGO samples. TEM microstructure (**c**) and (**d**) corresponding SAD pattern of sampleCuGO5.

Analysing the XRD pattern of all Cu and CuGO coating samples revealed an identical FCC Cu phase (JCPD 00/001-1241) [19], with nearly no peak position shifting or width broadening regardless of the processing voltage change for both Cu and GO/Cu samples. as shown in Figure 5a,b. However, a weak peak at 2 theta of 37° was noticed (marked ‘?’ in Figure 5b) for CuGO5 and CuGO6 samples, which could be indexed to (111) of Cu_2_O (JCPD 01-078-2076). TEM observation on the coating samples revealed that the average grain sizes of the coatings were in the range of 5–10 nm, indicating a nano-crystalline coating structures, as evidenced in Figure 5c. The selected area diffraction (SAD) pattern from the coating shows a strong set of fcc rings corresponding to Cu, while the weak rings correspond to Cu_2_O phase, confirming the tentative index of the XRD peak at 2 theta of 37°.

### 3.3. Tribological Properties of the Coatings

**Figure 6:**
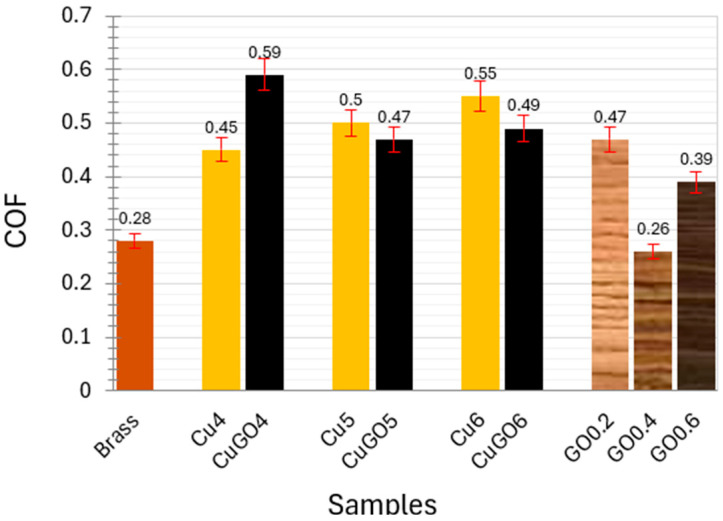
Coefficient of friction (COF) of Cu and CuGO samples compared with brass sample.

As the coating thickness of CuGO2 and CuGO3 samples was very thin (Figure 4), only coating samples produced under voltages of 4, 5 and 6 V were selected for the tribology tests. Figure 6 shows the coefficient of friction (COF) of the tested samples compared with the brass sample. It can be seen that all coating samples (Cu and CuGO) show a higher COF than that for the brass sample, while for Cu and CuGO samples, the GO-reinforced CuGO samples possess marginally lower COF, except for CuGO4. The last three columns in Figure 6 depict the COF of the Group 2 samples deposited under 5 V (with varying GO concentrations), and it can be seen that the coating plated with a GO concentration of 0.4 g/L revealed even slightly lower COF than that for the brass sample.

The surface microhardness of the bulk brass was measured to be about 143 HV_0.01_ and the hardness of the Cu and CuGO coating samples was 242 HV_0.01_ ± 39, which is slightly harder than the base material, brass. Hardness data analysis shows that the addition of GO and its quantity have no appreciable effect on coating hardness, although a marginal increase was observed for some of the coating samples.

**Figure 7:**
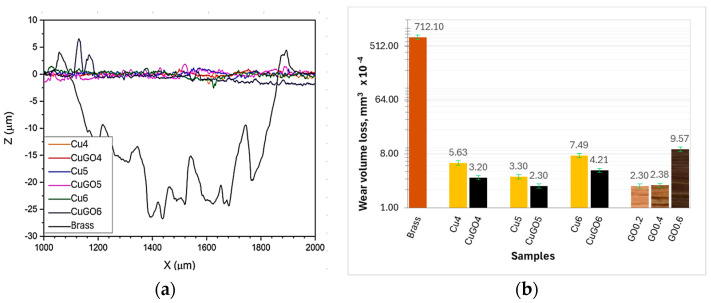
(**a**) 2D scanning of the wear tracks, and wear volume loss of (**b**) Group1 and 2 coating samples, comparing with brass substrate.

Although the coating hardness is only moderately higher than the brass ones, and the GO addition did not show a significant increase in the hardness of the GO-reinforced CuGO samples, as compared with the Cu sample, the wear performances of the GO-reinforced CuGO coatings exhibited substantial improvement. As can be seen from Figure 7a,b, all the coatings demonstrated significantly increased wear resistance by two orders of magnitude higher than the brass sample.

**Figure 8:**
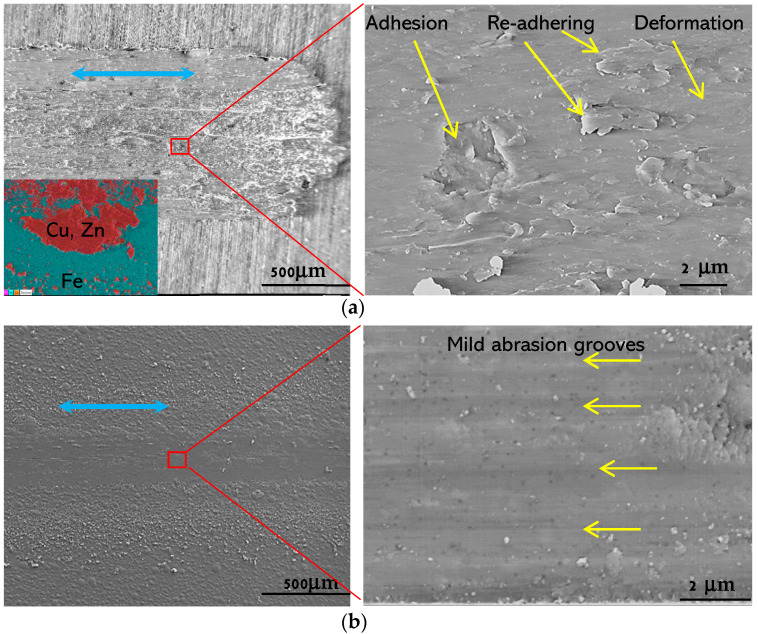
Typical wear tracks of brass and coating samples: (**a**) Brass (in insertion, EDX mapping showing brass transferred to the count-part stainless steel ball surface); (**b**) coating CuGO5. Sliding direction is shown by blue arrows.

Wear tracks on the brass and coating samples were observed under SEM. As can be seen from Figure 8a, the brass worn surface shows typical tearing/adhesion pits, severe plastic deformation and re-adhering materials from the count-part ball where wear debris was stuck (see insertion of Figure 8a). However, for all coating samples, the wear surfaces were relatively smooth and clean, and fine abrasion grooves were observed along the sliding directions, as shown in Figure 8b, a typical wear track of the coating samples.

Comparing the performance of the coating samples revealed that the CuGO samples can improve wear resistance to the extent of 1/3–2 times compared to Cu coatings, and the CuGO5 sample performed the best in terms of wear resistance under the current wear conditions (Figure 7b). As described in Section 2.2, Group 2 samples (Table 1) were designed based on this outcome, which was plated with a fixed voltage of 5 V and varying the GO concentrations in Cu solution from 0.2 to 0.6 g/L. Comparing the wear volume loss for the Group 2 samples, it can be seen that the GO content of 0.6 g/L in Cu solution reduced the wear resistance of the coating with volume loss four times that of the CuGO coating plated with GO content 0.2 and 0.4 g/L (Figure 7b).

## 4. Discussion

### 4.1. Tribological Property Improvements of Cu and CuGO Coatings

Compared with the brass samples, the Cu and CuGO coating samples show a higher friction coefficient (Figure 6) and moderately higher hardness than the brass sample, whereas the wear resistance of the coatings is significantly improved by two orders of magnitude compared to the brass samples (Figure 7a,b). This strong improvement in the wear behaviour could be associated with a different character of plastic deformation for Cu and brass. Rapoport etc. [20] reported that plastic deformation of Cu during wear is localised in thin surface layers about few microns, while it is close to 20 μm for brass. As evidenced in Figure 8a, the worn surface of brass is characterised by severe plastic deformation, which is attributed to the low stacking faults energy (14 mJ/m^2^) of the brass [21], and such deformation leads to the formation of thick and large wear particles, compared with the sticking and re-adhering, and therefore, to a high wear rate. For the Cu and CuGO coatings, very shallow abrasive grooves were observed with no perceptible adhesive wear features. It is the thinner plastic deformation layer of the Cu than brass, combined with the nano-crystalline structure of the Cu coatings, that might have contributed to the improved wear resistance of the coating samples. During the sliding, when the plastic deformation concentrated within the surface layer, deformation hardening could occur within the deformed thin layer and the nanocrystalline structure served abnormal dislocation and twin originates, which made the hardening effect further and provided low wear. This assumption is supported by the research of Meshi [22], who found that the friction of Cu is accompanied by a localised deformation at the thin surface. This resulted in the formation of a nanocrystalline structure with an improved surface hardness and provided low wear of Cu samples.

**Figure 9:**
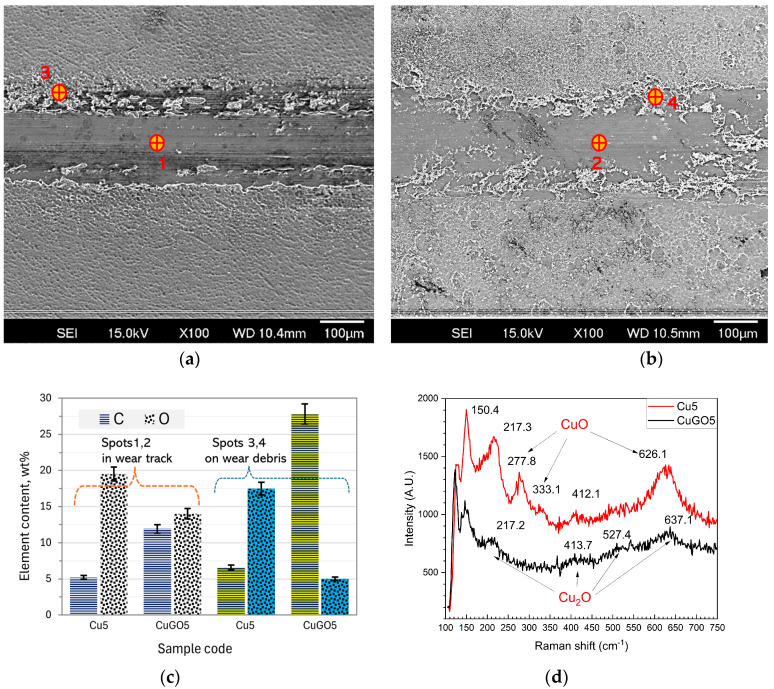
SEM images of wear tracks from (**a**) Cu5 and (**b**) CuGO5 samples. (**c**) EDX composition (carbon and oxygen only) analysis from wear tracks (denoted as 1, 2 in (**a**,**b**)) and wear debris (denoted as 3, 4 in (**a**,**b**), and (**d**) Raman spectra from wear tracks of Cu and CuGO5 samples.

Comparing the wear properties of the coating samples, the GO embedded (0.2–0.4 g/L) CuGO samples had better wear resistance than the Cu coating samples (Figure 7) to the extent of 1/3–2 times. This improvement could be attributed to the solid lubricant of GO sheets [16]. Firstly, GO films acted as a lubricant film by separating the count-parts contact at the tribological testing zone and decreasing the occurrence of adhesive wear. This is evidenced by the higher carbon content of the CuGO5 sample than the Cu5 sample in both the wear track and the wear debris areas (Figure 9a–c). This is further supported by the lower CoF of most CuGO coatings compared to the corresponding Cu samples (Figure 6). Secondly, embedded GO sheets changed oxidation behaviour or oxidative wear of the coatings during sliding. Surface morphology images coupled with chemical element analysis, shown in Figure 9a–c, indicated a lower oxygen content detected from the CuGO5 sample than from the Cu5 sample, both in the wear track and in the debris areas. Raman analysis confirmed the formation of cupric oxide CuO and cuprous oxide Cu_2_O [23] within the Cu coating samples. Only low-intensity peaks of cuprous oxide Cu_2_O were detected for the CuGO5 sample (Figure 9d), consistent with the TEM observation shown in Figure 5d. This reduced oxidation behaviour of the CuGO sample is believed to have resulted from GO films, which reduced the coefficient of friction and thus reduced the friction heat on the surface [18]. Less oxides within the wear debris further reduced the wear loss of the CuGO samples.

### 4.2. Optimal Brush Plating Conditions for Wear Resistance Coatings

It is clear that the wear performance of Cu and CuGO coatings produced by brush plating outperformed the brass material. Among the CuGO coatings, their wear performance is related to the brush plating voltage and the amount of GO additions in the Cu plating solution. From a processing voltage point of view, the 5 V processed coatings possess the lowest wear volume loss (Figure 7). When CuGO coatings are produced under this optimal coating processing voltage, the wear performance of the coatings is related to the GO quantity. A low quantity (0.2–0.4 g/L) of GO addition to Cu coatings enhances their wear performance. A further increase in the GO to above 0.4 g/L in the Cu solution leads to reduced wear resistance. This may be related to the density of the coatings. As the GO films are embedded within the lamellae layers (Figure 3d), when too many GO films are introduced, the bounding between the GO films is loosely connected, which will reduce the density of the coatings, resulting in severe wear loss.

To sum up the above, for the best wear-resistant Cu coatings, the optima brush plating conditions are at 5 V with GO addition at the concentration of 0.2–0.4 g/L in the plating solution.

## 5. Conclusions

In this work, an electro-brush plating technique was employed for the novel fabrication of a copper-graphene oxide (GO) nano-composite coating and the following conclusions can be drawn.

- The research demonstrated the fabrication of thick (>19.2 μm) GO/Cu composite coatings with good adhesive to the substrate on brass by brush plating under voltage equal to and higher than 4 V.
- The detailed characterisation of the brush-plated coating by SEM, EDX and GDOES revealed that GO can be successfully introduced across the whole composite coating layer.
- The GO/Cu composite coatings produced under 5 V (GO/Cu5) and 6 V (GO/Cu6) can reduce friction and increase the wear resistance by two orders of magnitude compared to brass, mainly due to the self-lubricating of the GO added into the coatings.

The novel self-lubricating and wear-resistant Cu/GO composite coating developed from this research could pave the way towards the potential application for brass sliding bearings.

## Figures and Tables

**Table 1:** Electro-plating procedures for applying Cu coating and GO/Cu composite coatings on brass.

No.	Operation	Solution	Voltage/V	Time/min
1	Electrical active-clean	Active-cleaning solution	7	3
2	Nickel strike	Special nickel solution	7	3
3	Final plating	Cu or Cu + GO solutions (0.2–0.6 g/L)	2–6	30

**Table 2:** Sample code and corresponding coating parameters for GO/Cu and Cu coating samples.

Group	Sample Code		Plating Solution		Plating Voltage/V
	Cu	CuGO	Cu	CuGO	
1	Cu2	CuGO2	Cu	Cu + GO (0.2 g/L)	2
	Cu3	CuGO3			3
	Cu4	CuGO4			4
	Cu5	CuGO5			5
	Cu6	CuGO6			6
2	GO0.2		Cu + GO (0.2 g/L)		5
	GO0.4		Cu + GO (0.4 g/L)		
	GO0.6		Cu + GO (0.6 g/L)		
	GO0.8		Cu + GO (0.8 g/L)		

## Data Availability

Data are contained within the article.
